# Multidisciplinary approach for treatment of a dentigerous cyst – marsupialization, orthodontic treatment, and implant placement: a case report

**DOI:** 10.1186/s13256-018-1829-2

**Published:** 2018-10-10

**Authors:** Noriaki Aoki, Kazuma Ise, Arisa Inoue, Yasufumi Kosugi, Chika Koyama, Masaki Iida, Junichi Baba, Toshinori Iwai, Kenji Mitsudo

**Affiliations:** 1 Department of Oral and Maxillofacial Surgery, Saiseikai Yokohamashi Nanbu Hospital, 3-2-10 Konandai Konanku, Yokohama City, Kanagawa Japan; 2 Department of Oral and Maxillofacial Surgery, Yokohama City University Graduate School of Medicine, Yokohama City, Kanagawa Japan

**Keywords:** Dentigerous cyst, Multidisciplinary approach, Implant, Orthodontic traction, Marsupialization, Orthodontic treatment

## Abstract

**Background:**

Dentigerous cysts are common odontogenic cysts associated with unerupted teeth. We describe a previously unreported case of a multidisciplinary approach using surgical, orthodontic, and implant treatment to establish the occlusion for a patient with a maxillary dentigerous cyst.

**Case presentation:**

An 18-year-old Japanese woman visited our hospital with a chief complaint of gingival swelling in her anterior maxillary region, midline diastema, and tooth crowding. Her main symptom was this gingival swelling. A panoramic radiograph revealed a radiolucent area, 30 mm in diameter, round in shape, and with well-demarcated margins including the maxillary canine. Computed tomography revealed a cystic cavity filled with homogeneous fluid of the same density as water, and a distolingually inclined canine. Our clinical diagnosis was maxillary dentigerous cyst with an unerupted distolingually inclined canine. The selected treatment was marsupialization of the dentigerous cyst, followed by orthodontic traction of the unerupted canine, and simultaneous orthodontic treatment of the midline diastema and tooth crowding. The orthodontic traction failed because the canine did not erupt completely, and the canine was extracted. The treatment plan was then changed to implant treatment after the tooth crowding and midline diastema had been improved. Because the alveolar ridge width was inadequate, the implant was placed after a two-stage implant treatment; therefore, a satisfactory occlusion could be achieved. Our patient did not experience any complications, and the cyst has not recurred. A radiograph taken 7 years after marsupialization of the dentigerous cyst revealed that the cystic cavity had been replaced by new bone.

**Conclusions:**

In general, orthodontic traction of an unerupted tooth after marsupialization should be the best option. However, if orthodontic traction fails, a multidisciplinary approach involving implant treatment may be necessary. We describe a case in which a multidisciplinary approach involving surgical, orthodontic, and implant treatment was used to establish a satisfactory occlusion for a patient with a dentigerous cyst.

## Background

Dentigerous cysts are the most common odontogenic cysts of the jaws, and sometimes inhibit the eruption of teeth [1–3]. The conventional treatment plan is cyst removal and marsupialization. Marsupialization therapy can be useful to promote the spontaneous eruption of the involved tooth within the cyst. However, tooth eruption does not always occur spontaneously after marsupialization [4]. The unerupted tooth may need to be extracted, leaving insufficient alveolar bone for implant placement [3]. In such cases, dentigerous cysts of the jaw present a challenge in establishing the occlusion because a multidisciplinary approach is required that includes prosthetics or implant treatment. We used orthodontic traction combined with marsupialization therapy to treat a dentigerous cyst associated with a deeply impacted maxillary canine. However, this approach did not result in eruption of the tooth; therefore, implant treatment was performed instead. The purpose of this case report is to emphasize the success of a multidisciplinary approach to managing a dentigerous cyst and stimulating new bone formation in the surgical field after marsupialization.

## Case presentation

**Fig. 1:**
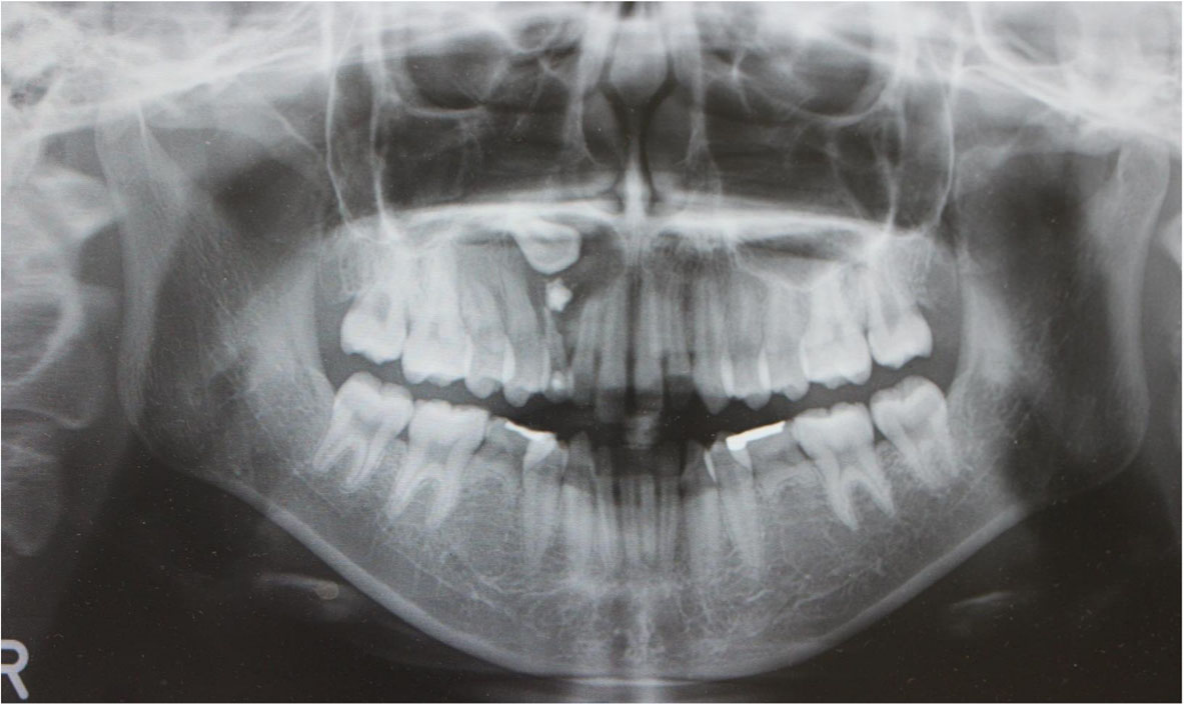
Panoramic radiograph at initial visit. Water-soluble root canal agent seen

**Fig. 2:**
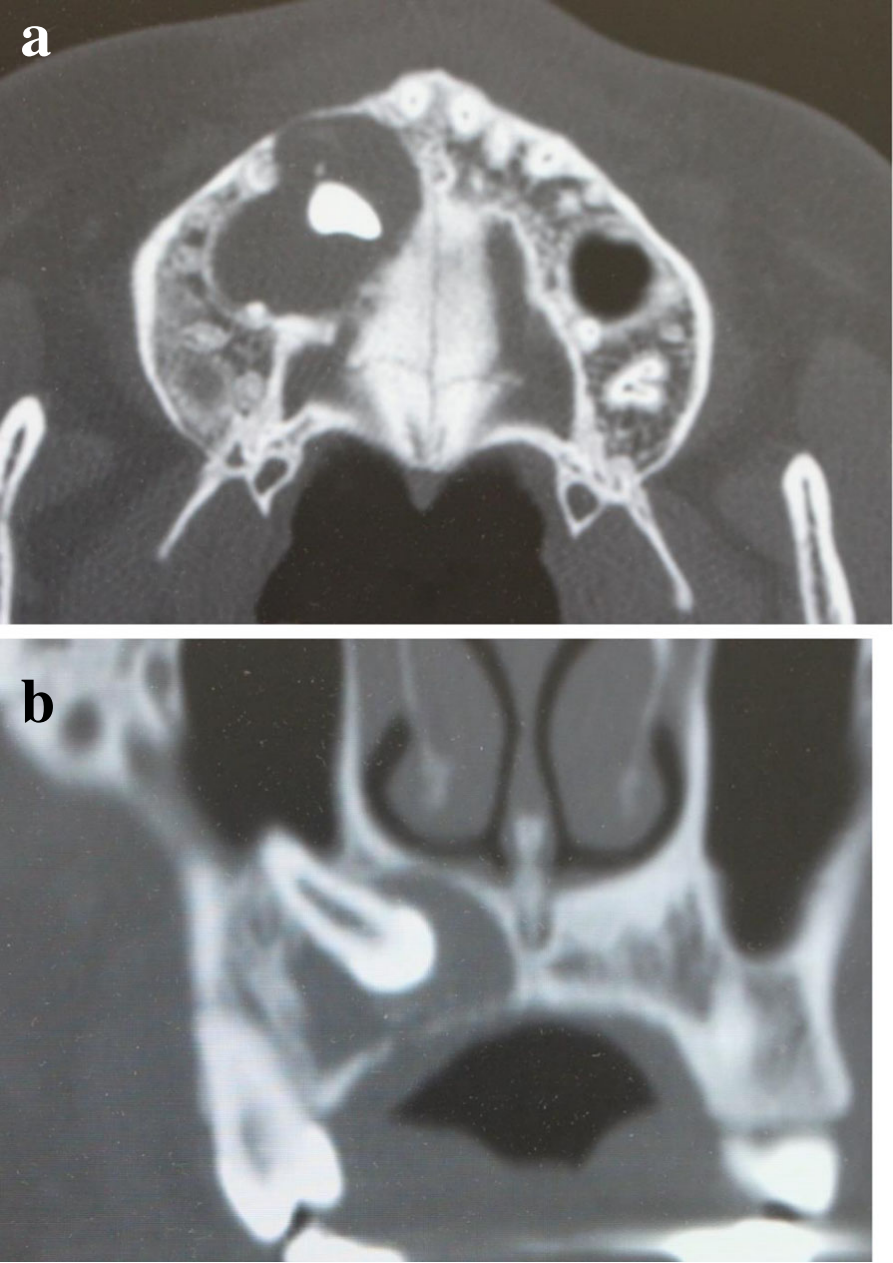
Computed tomography at initial visit. **a** Axial image. **b** Frontal image

**Fig. 3:**
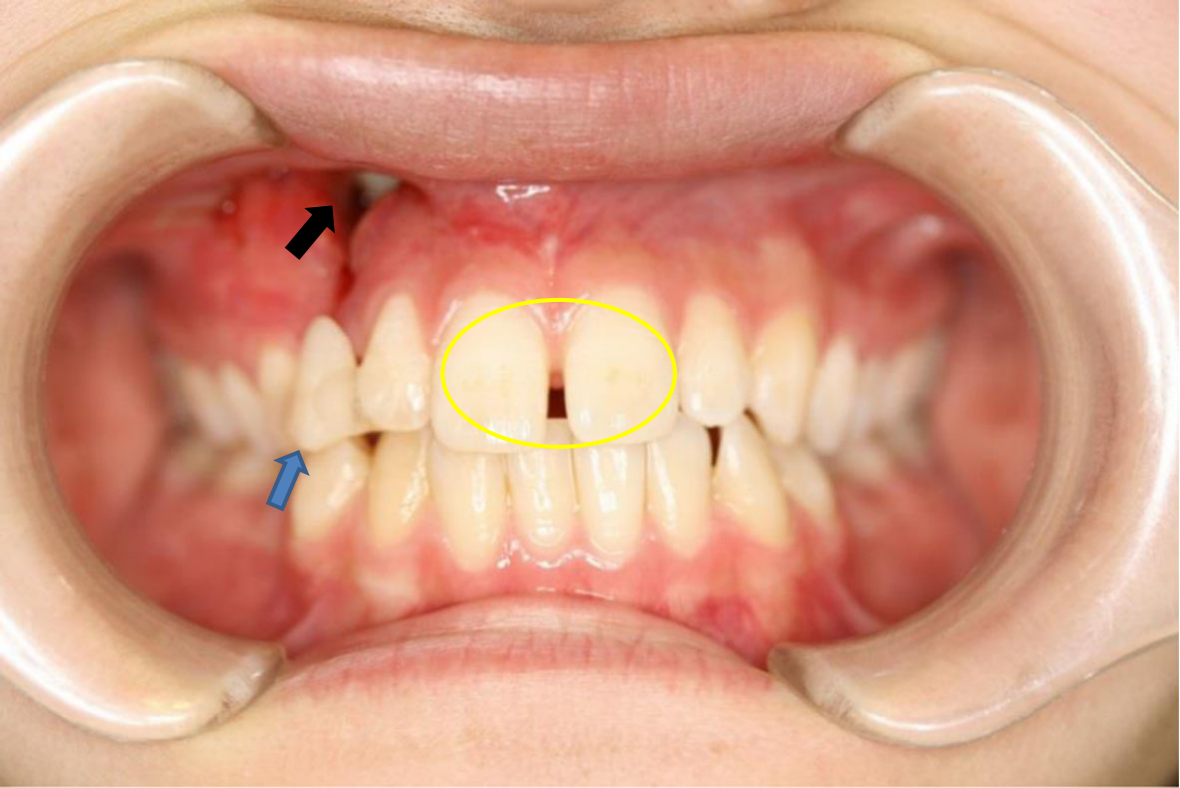
Marsupialization (*black arrow*), midline diastema (*yellow circle*), and tooth crowding. Temporary crown placed to cover the space (*blue arrow*)

**Fig. 4:**
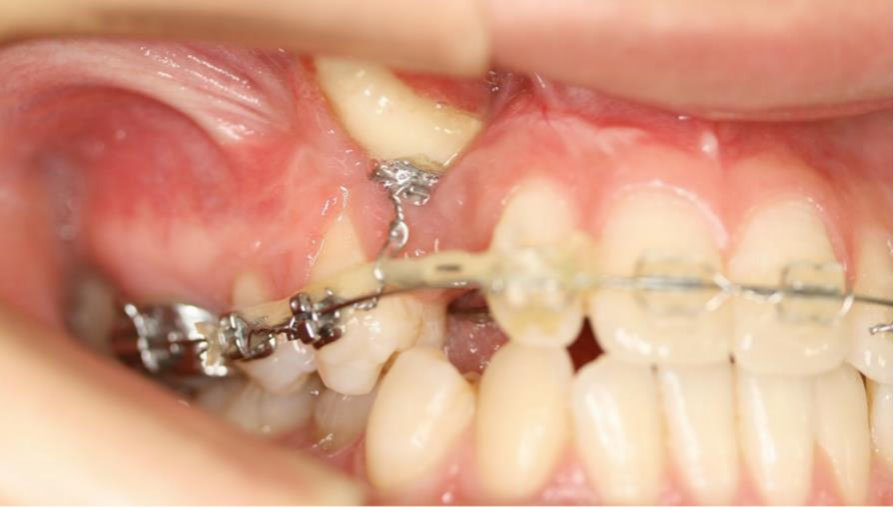
Orthodontic treatment performed for traction of unerupted canine and improvement of midline diastema and tooth crowding

**Fig. 5:**
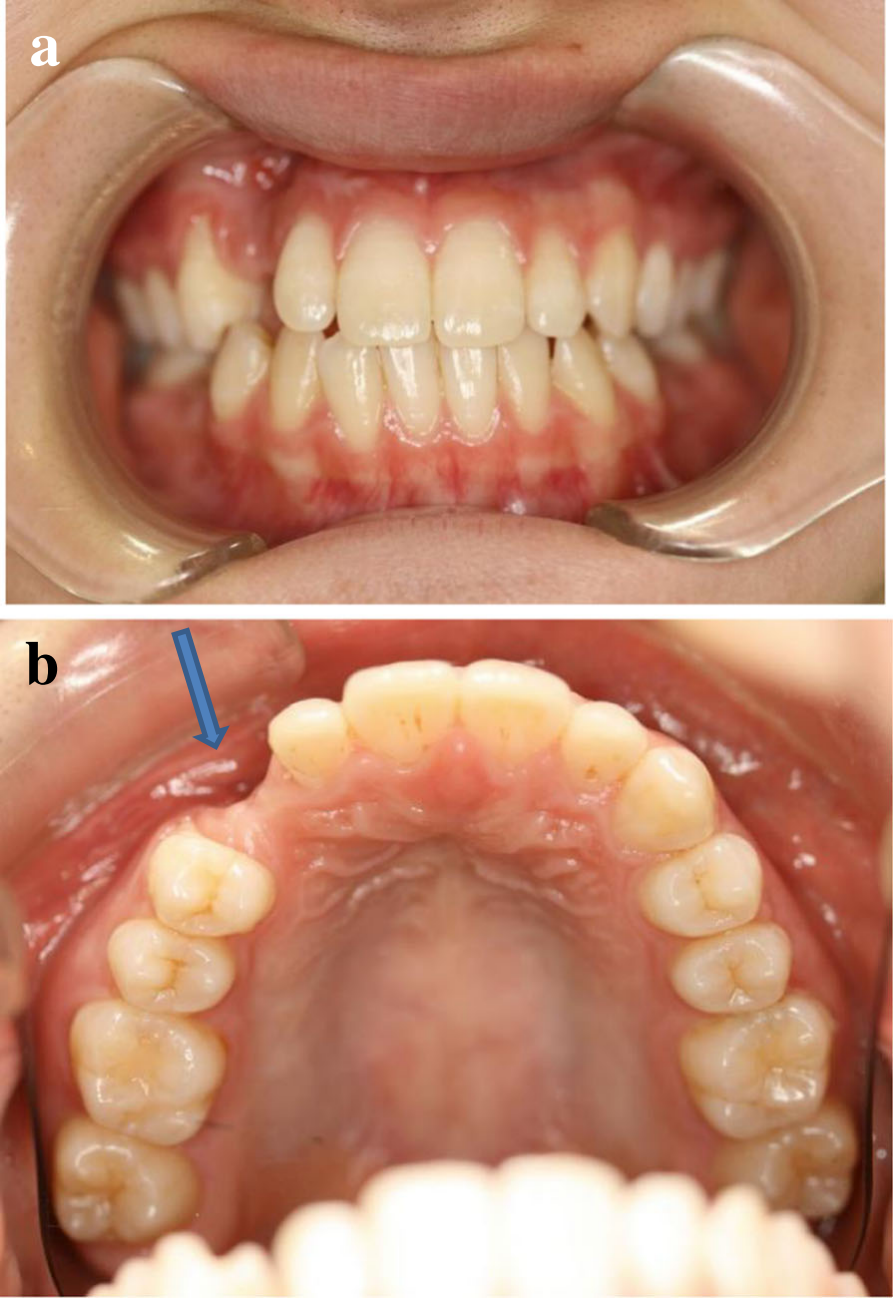
Orthodontic treatment has finished after canine extracted. Tooth crowding and midline diastema have improved. **a** Frontal view. **b** Occlusal view. Less bone width (*blue arrow*)

**Fig. 6:**
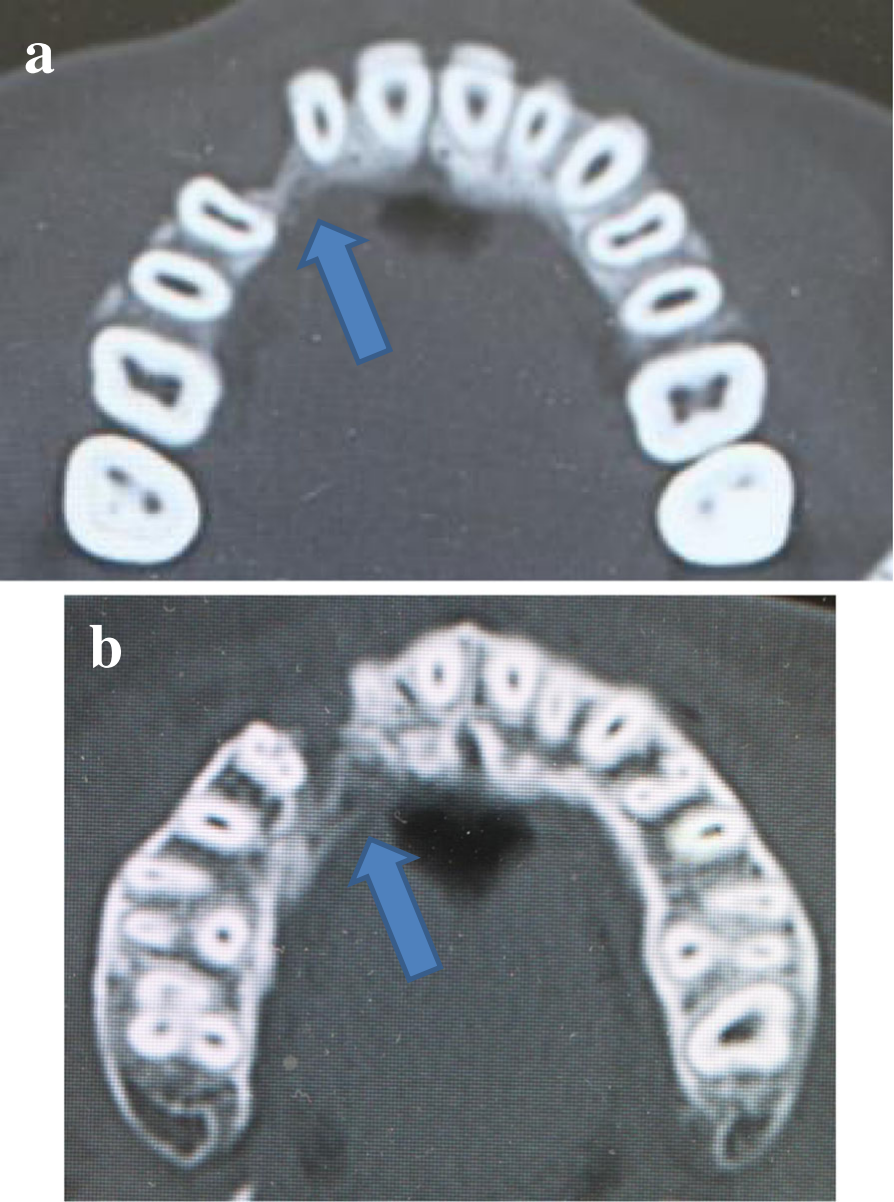
Insufficient bone to install implant (*blue arrow*) on computed tomography, before implant placed. **a** Less bone width at the alveolar ridge. **b** Less bone width at their deep area

**Fig. 7:**
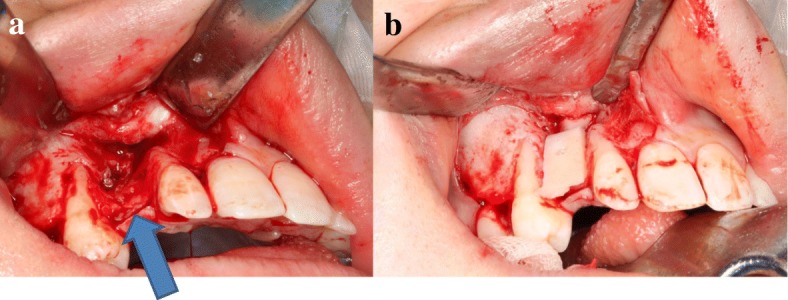
Bone augmentation to widen the bone width. **a** Less bone width (*blue arrow*). **b** Autogenous bone harvested from the mandibular ramus placed on the surface of defect area

**Fig. 8:**
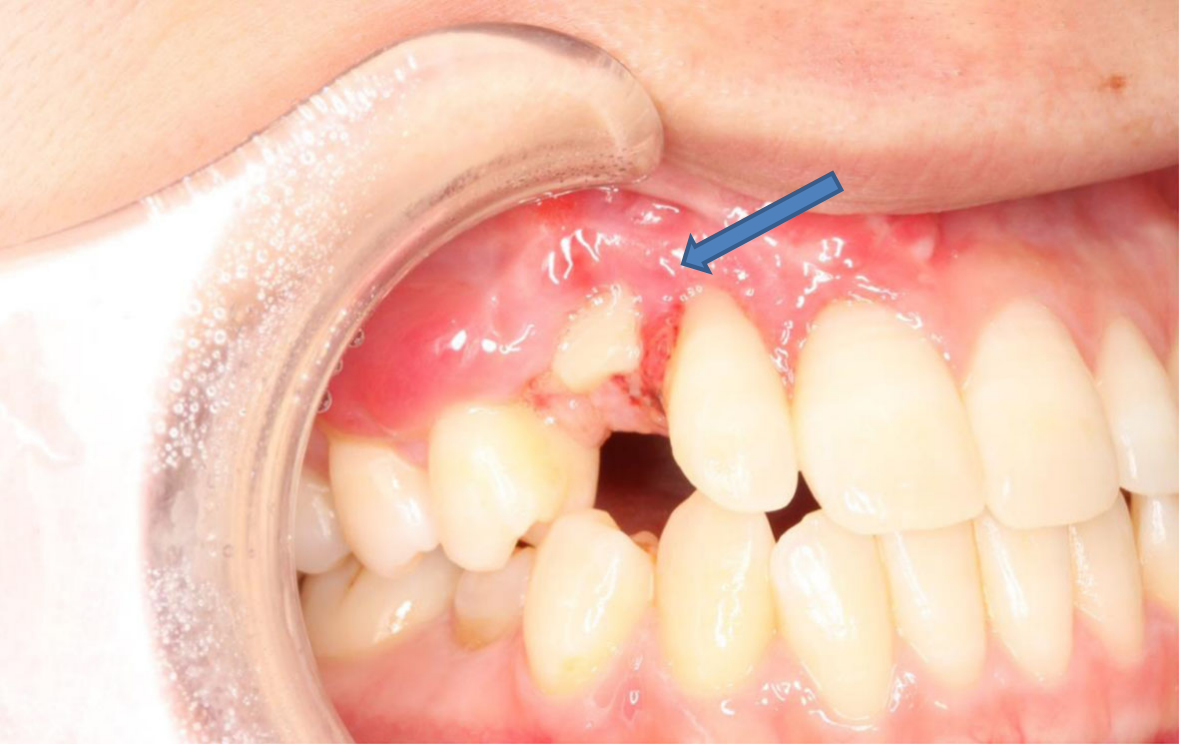
On the way to the second operation, a part of the bone (*blue arrow*) exposed

**Fig. 9:**
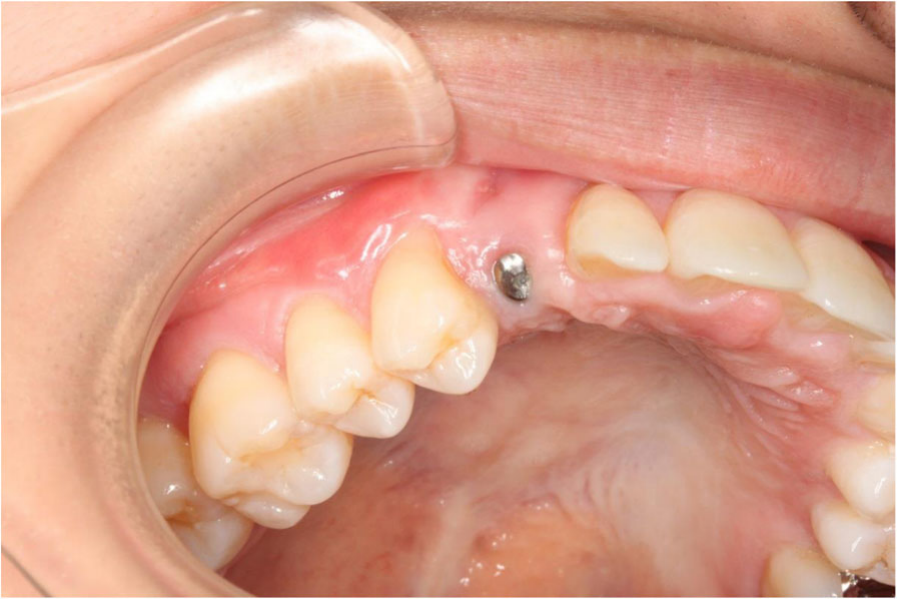
Eleven months after bone graft, implant placed

**Fig. 10:**
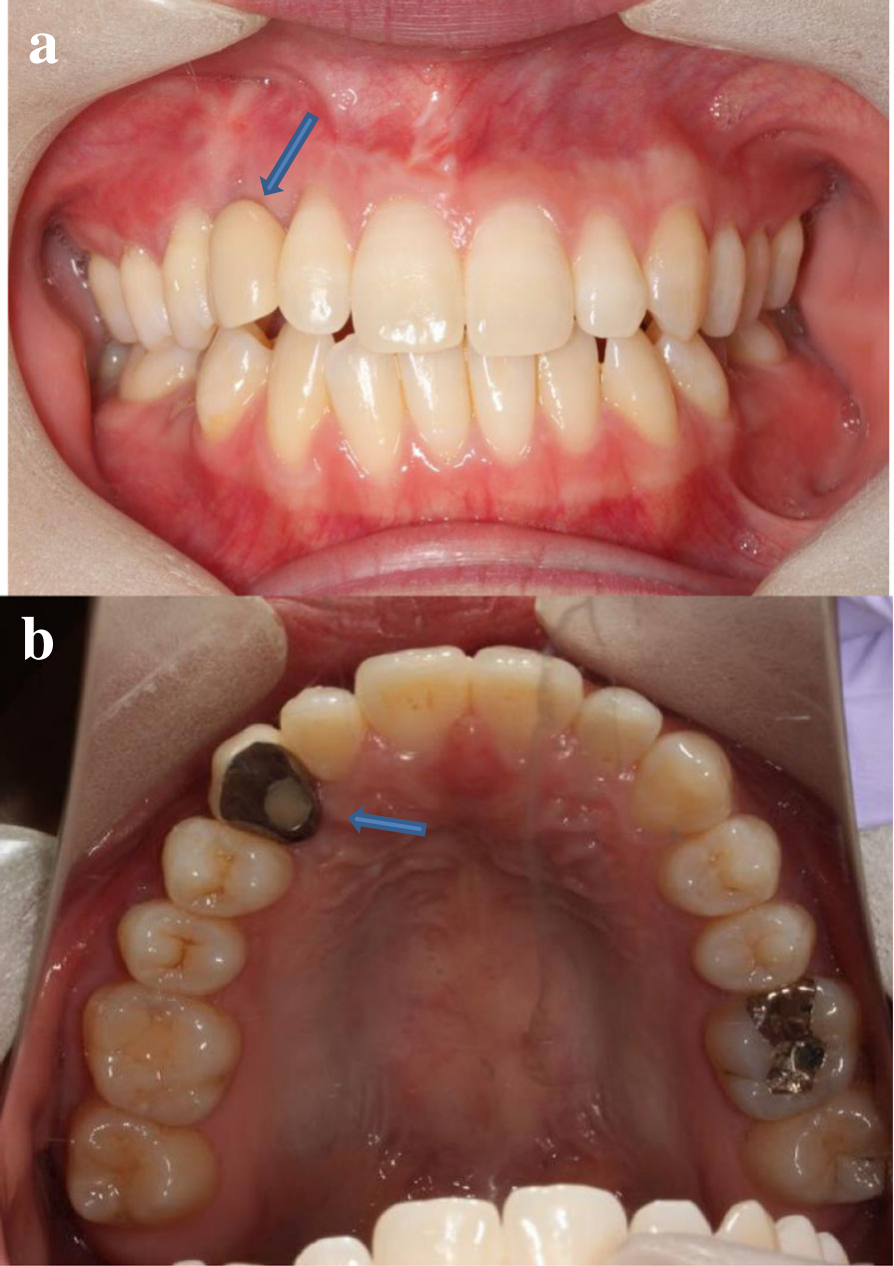
Final occlusion showing good clinical results. **a** Frontal view. **b** Occlusal view. Implant prosthesis (*blue arrow*)

**Fig. 11:**
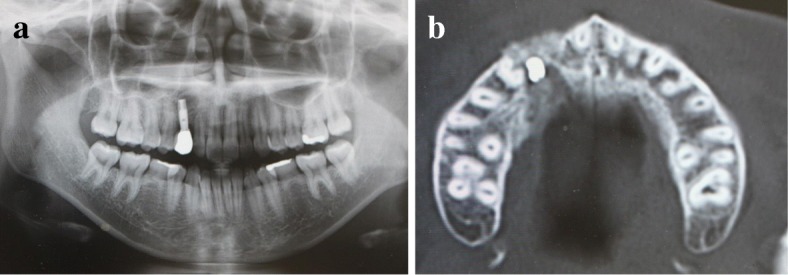
Follow-up image. **a** Implant and suprastructure. **b** Sufficient bone around implant seen

**Fig. 12:**
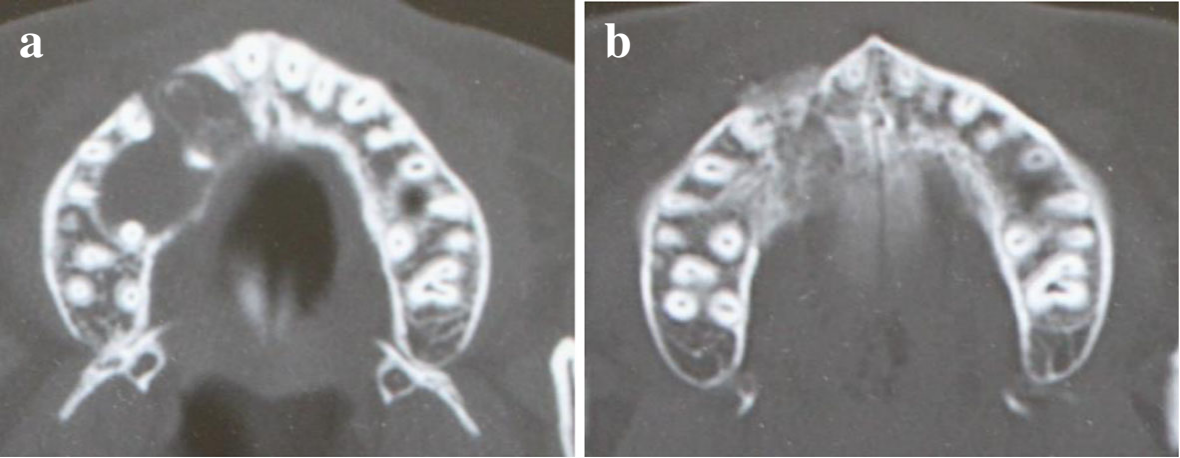
**a** and **b** Comparison between preoperative and postoperative computed tomography

An 18-year-old Japanese woman visited our hospital with a chief complaint of gingival swelling in the region of her upper right anterior teeth, midline diastema, and tooth crowding. Her main symptom was gingival swelling in the region of her upper right anterior teeth. She had no medical, family and psychosocial history, and she had not undergone relevant past interventions. A physical examination showed no problems. She had previously undergone root canal treatment for the remaining deciduous canine because of pus discharging from the root canal. An intraoral examination revealed a diffuse swelling around the deciduous canine, midline diastema, and tooth crowding. A panoramic radiograph revealed a round radiolucency with a diameter of 30 mm, with well-demarcated margins around the maxillary canine (Fig. 1). Computed tomography (CT) revealed that the cystic cavity surrounded the maxillary canine and was filled with a homogeneous water-like fluid with density (Fig. 2). Our clinical diagnosis was maxillary dentigerous cyst with an unerupted maxillary canine. Marsupialization was carried out under general anesthesia, and the unerupted canine was left in place (Fig. 3). A histopathological examination revealed the diagnosis of dentigerous cyst. However, the marsupialization did not result in eruption of the canine. Three months later, orthodontic traction was applied to the unerupted canine, and simultaneously orthodontic treatment to correct the tooth crowding and midline diastema (Fig. 4). The orthodontic traction of the maxillary canine failed, and the canine was then extracted. On the other hand, crowding and midline diastema were improved (Fig. 5). The revised treatment plan was to undertake staged implant placement, because the alveolar bone at the implant site was inadequate, 2 mm alveolar width on CT (Fig. 6). We were planning to bone graft after the mucosa completely healed up because severe scar tissue caused by the previous marsupialization was seen in the canine tooth extraction area. Bone augmentation was performed with an autogenous bone graft that was harvested from the mandibular ramus to widen the alveolar bone (Fig. 7). While doing the implant placement in the second operation, a part of the grafted bone was exposed, and was trimmed with a bur, several times (Fig. 8). The wound had completely healed up in 6 months. For her busy schedule, 11 months after the bone graft, the implant was inserted without any problems. The implant was uncovered, and the abutment was connected under local anesthesia (Fig. 9). The occlusion was stabilized by the implant, following a screw-retained prosthodontic procedure performed with appropriate implant stability. The occlusion was successfully restored by the insertion of the implant (Table 1). Good clinical results were achieved with no severe complications or recurrence of the cyst (Fig. 10). Her postoperative course was uneventful for 7 years. A CT scan taken 7 years after marsupialization showed that the cystic cavity had been replaced by new bone, and that the implant was stable in the surrounding bone (Figs. 11, 12).

**Table 1:** Timeline

	August, 2010	January, 2011	April, 2011	October, 2012	December, 2012	September, 2015	August, 2016	January, 2017	February, 2017
Time	T = 0	T = 5 months	T = 8 months	T = 26 months	T = 28 months	T = 61 months	T = 72 months	T = 77 months	T = 78 months
Event	Initial visit	Marsupialization	Orthodontic traction started	Unerupted canine extraction	Orthodontic removal retainer placed	Bone graft	Implant placed	Second surgery	Suprastructure placed

## Discussion

Dentigerous cysts are the most common of the odontogenic cysts. They often present as a localized swelling of the alveolar bone associated with an unerupted tooth. The most common sites are around the crown of the mandibular third molar, followed by the maxillary canine, maxillary third molar and, rarely, the maxillary central incisor [1, 2]. On radiographic examination, a dentigerous cyst usually appears as a well-demarcated unilocular radiolucency, surrounding the crown of an unerupted tooth [3]. In the present case, the cyst was located in the maxillary canine region and was associated with an unerupted maxillary canine.

Marsupialization is a surgical procedure that decreases the intracystic pressure and gradually shrinks the cyst. This procedure creates an opening in the cystic wall to drain the contents of the cyst, and occasionally the cyst wall is sutured to the oral mucosa [4]. The advantage of this procedure is that it stimulates the eruption of the cyst-associated tooth, with or without orthodontic traction. However, the disadvantages of marsupialization are the long postoperative duration of treatment and the discomfort of leaving the wound open [3]. In general, marsupialization and orthodontic traction are considered to be the best option for patients with a dentigerous cyst that includes an unerupted tooth. Orthodontic traction of the unerupted tooth is often performed after marsupialization, if there is adequate space for the unerupted tooth. Some studies have been conducted to determine the optimal timing of orthodontic traction to allow the unerupted tooth to erupt from the cyst. The findings were that spontaneous eruption of the cyst-associated tooth is predictable and may take place 3 months after marsupialization; however, orthodontic traction may be required for longer duration in some cases [3, 5]. The predictive factors for the eruption of a cyst-associated tooth after marsupialization are controversial. Some authors reported that many factors influence tooth eruption, including the age of the patient, tooth depth, tooth inclination, stage of root formation (open or closed apex), and amount of space available [6, 7]. In contrast, others reported that these factors are not significant and do not affect tooth eruption [8]. In this case, given that our patient was 18-years old and root formation was complete, marsupialization combined with orthodontic treatment was thought to be the most appropriate treatment. Originally, the treatment plan was marsupialization and tooth eruption with or without using orthodontic traction. The reason why the orthodontic traction failed is that the severely lingually tilted tooth axis of the canine prevented eruption of the canine. Given that the patient was 18-years old and concerned about esthetics, when this treatment failed, we considered the use of an implant after gaining informed consent.

Implant placement after marsupialization of a dentigerous cyst and bone grafting is not a well-documented procedure in the clinical literature. However, the grafting procedure is crucial, especially when there is a large defect to be filled [9]. Although marsupialization is a reliable procedure, there is a lack of information about implant placement after bone regeneration following marsupialization without bone grafting [10, 11]. We considered other ways to augment the alveolar ridge before implant placement, such as alveolar ridge split technique. In the present case, the alveolar bone was found to be inadequate after marsupialization of such a large cyst. Therefore, the alveolar ridge width was too narrow to perform alveolar ridge split technique, and standard technique using an autogenous bone graft was selected. In the first operation, an autogenous bone graft was placed in the area of the defect. Before the second operation, a part of the grafted bone was exposed because of severe buccal scarring resulting from the surgery. The bone was trimmed with a bur, and the implant was subsequently successfully placed with a lingual inclination in a completely healed area. This case highlights the need to consider all factors, such as the age of the patient, the surgically treated area, and the size and characteristics of the lesion, to achieve a successful result. The present case also demonstrates the success of a multidisciplinary approach to treating a large cyst in an 18-year-old patient, as well as augmenting new bone formation with an autogenous bone graft for the placement of an implant to achieve oral rehabilitation.

## Conclusions

When treating a dentigerous cyst involving an unerupted tooth, a multidisciplinary approach is necessary from beginning to end to achieve and establish proper occlusion. The present case demonstrates how multiple procedures such as surgery, orthodontic treatment, and implant placement led to a satisfactory outcome for the patient in terms of esthetics and occlusion. Occasionally, a timely switch of treatment plan and multidisciplinary treatment are needed to solve problems that may arise. Clinicians must work together with the surgeon, orthodontist, and implantologist to choose the best treatment for the patient.

## Abbreviation

CT: Computed tomography
